# Thrombin induced platelet-fibrin clot strength in relation to platelet volume indices and inflammatory markers in patients with coronary artery disease

**DOI:** 10.18632/oncotarget.19450

**Published:** 2017-07-22

**Authors:** Hai-Chen Lv, Hong-Yi Wu, Jia-Sheng Yin, Jun-Bo Ge

**Affiliations:** ^1^ Department of Cardiology, First Affiliated Hospital of Dalian Medical University, Dalian, China; ^2^ Shanghai Institute of Cardiovascular Diseases, Department of Cardiology, Zhongshan Hospital, Fudan University, Shanghai, China

**Keywords:** coronary artery disease, thrombelastography, inflammatory marker, platelet volume index, platelet activation

## Abstract

Platelet aggregation and inflammation are both implicated in coronary artery disease (CAD). Thrombin induced platelet-fibrin clot strength (MA_Thrombin_) measured by thrombelastography (TEG) has been proved to be a novel marker of platelet aggregation. The aim of this study was to investigate the correlation of MA_Thrombin_ to platelet volume indices (PVIs) or to inflammatory markers in different types of CAD. 206 patients with different types of CAD were enrolled. MA_Thrombin_, PVIs, including mean platelet volume (MPV), platelet distribution width (PDW), and platelet-large cell ratio (P-LCR) as well as inflammatory markers, including high-sensitivity C-reactive protein (hs-CRP) and fibrinogen (Fbg) were measured. Multiple linear regression models were used to analyze the association between MA_Thrombin_, PVIs, and inflammatory markers. MA_Thrombin_ and inflammatory markers both varied with CAD types (P<0.001). MA_Thrombin_ was correlated to PVIs in NSTEMI individuals (MPV, r=0.393, P=0.007; PDW, r=0.334, P=0.023; P-LCR, r=0.382, P=0.008), but had inner-link with inflammatory markers in STEMI cases (hs-CRP, r=0.499, P<0.001; Fbg, r=0.500, P<0.001). These findings may suggest different mechanisms of platelet aggregation in different types of CAD. Moreover, MA_Thrombin_ may be used as a potential parameter to evaluate platelet aggregation and inflammation together.

## INTRODUCTION

Platelet aggregation plays an important role in the pathogenesis of coronary artery disease (CAD) [1–4], but bedside monitoring of platelet aggregation is challenging clinically. Recently, thrombin induced platelet-fibrin clot strength (MA_Thrombin_) measured by thrombelastography (TEG) has been suggested as a novel marker to identify platelet hyperaggregability in patients with acute myocardial infarction (AMI) [5]. In addition, previous studies have demonstrated that MA_Thrombin_ could provide prognostic value on high platelet reactivity (HPR) after anti-platelet therapy [6–8]. However, the underlying determinants of MA_Thrombin_ remain unclear.

Previous studies have indicated that platelet volume indices (PVIs), such as mean platelet volume (MPV), platelet distribution width (PDW), and platelet-large cell ratio (P-LCR), could elevate in patients with acute coronary syndrome (ACS), which might due to larger platelets containing more pro-aggregating mediators and presenting more enhanced functions [9–11]. From this view, PVIs, perhaps, can also be regarded as a measurement of platelet aggregation. Still, the inner-link between MA_Thrombin_ and PVIs in CAD patients remains confusing. Otherwise, inflammatory markers, such as high-sensitivity C-reactive protein (hs-CRP) and fibrinogen (Fbg), have been confirmed to be associated with the occurrence and poor prognosis of ACS tightly [12–14]. But whether there exists any relationship between MA_Thrombin_ and inflammatory markers is controversial either.

The aim of this study was to detect the magnitude of MA_Thrombin_ and its relationship with PVIs and inflammatory markers in patients with different clinical types of CAD.

## RESULTS

### Patient demographics

Baseline characteristics of all the participants are illustrated in Table 1, according to different clinical type of CAD. There was no statistical difference about age, sex, tobacco use, body mass index (BMI), history of diabetes, dyslipidemia, and stroke in each group, but the prevalence of hypertension varied (P=0.024). Both aspirin and anti-coagulation application were significantly different in each group (P<0.001), however, prescriptions of statins, β-blockers, and proton pump inhibitors (PPIs) were demonstrated equally between groups. Moreover, patients with myocardial infarction tended to present higher white blood cell count (WBC), fasting blood glucose, and N-terminal pro-brain natriuretic peptide (NT-proBNP), as well as lower left ventricular ejection fraction (LVEF).

**Table 1:** Demographics of study population according to clinical presentation

	Overall	STEMI	NSTEMI	UAP	SAP	P-value
	N=206	N=67	N=50	N=33	N=56	
**Baseline characteristic**						
Age	62.99±11.01	64.60±11.53	62.98±12.05	63.70±8.57	60.66±10.56	0.255
Male(N,%)	156(75.7)	51(76.1)	39(78.0)	24(72.7)	42(75.0)	0.955
Smoking(N,%)	110(53.4)	41(61.2)	28(56.0)	18(54.5)	23(41.1)	0.157
BMI(kg/m^2^)	24.89±1.48	24.67±1.29	24.89±1.51	24.89±1.73	25.16±1.52	0.338
Hypertension(N,%)	130(63.1)	34(50.7)	39(78.0)	20(60.6)	37(66.1)	**0.024**
Diabetes mellitus(N,%)	72(35.0)	28(41.8)	19(38.0)	12(36.4)	13(23.2)	0.171
Dyslipidemia(N,%)	43(20.9)	12(17.9)	15(30.0)	6(18.2)	10(17.9)	0.343
Stroke(N,%)	16(7.8)	5(7.5)	4(8.0)	2(6.1)	5(8.9)	0.969
**Medication history**						
Aspirin(N,%)	57(44.2)	10(18.2)	15(39.5)	13(81.2)	19(95.0)	**<0.001**
Anti-coagulation(N,%)	143(69.4)	66(98.5)	47(94.0)	19(57.6)	11(19.6)	**<0.001**
Statins(N,%)	204(99.0)	67(100.0)	50(100.0)	32(97.0)	55(98.2)	0.393
β-blockers(N,%)	189(91.7)	61(91.0)	45(90.0)	31(93.9)	52(92.9)	0.909
PPI(N,%)	57(27.7)	30(44.8)	21(42.0)	1(3.0)	5(8.9)	**<0.001**
**Other characteristics**						
WBC(×10^9/L)	7.52±2.76	9.44±3.48	7.48±1.80	6.24±1.56	6.03±1.34	**<0.001**
Platelet(×10^9/L)	198.07±53.09	206.15±60.67	197.82±48.22	182.12±44.00	198.04±51.56	0.210
Fasting blood glucose(mmol/L)	6.32±2.35	7.41±2.88	6.34±2.06	5.89±2.30	5.25±0.98	**<0.001**
Serum creatinine(mmol/L)	76.21±20.66	75.23±21.89	75.24±16.96	82.03±29.35	74.82±15.37	0.374
NT-proBNP(ng/L)	931.13±1459.31	1449.45±1608.49	1250.59±1903.66	610.68±710.20	216.82±551.75	**<0.001**
Lp(a)(mg/L)	209.30±201.45	165.03±149.96	240.45±210.04	246.94±276.08	213.95±193.31	**0.136**
LVEF(%)	62.08±9.22	57.17±9.43	63.68±7.62	61.31±9.43	66.94±7.02	**<0.001**

Data are expressed as mean ± standard deviation(SD) or number of patients (percentage).

STEMI, ST segment elevation myocardial infarction; NSTEMI, non-ST segment elevation myocardial infarction; UAP, unstable angina pectoris; SAP, stable angina pectoris; BMI, body mass index; PPI, proton pump inhibitor; WBC, white blood cell; NT-proBNP, N-terminal pro-brain natriuretic peptide; Lp(a), Lipoprotein(a); LVEF, left ventricular ejection fraction.

### Correlation of TEG measured MA_Thrombin_ and CAD types

As displayed in Table 2, there were significantly statistical differences in the magnitudes of MA_Thrombin_ among groups (P=0.026). Moreover, MA_Thrombin_ appeared to be higher in patients with AMI (including ST segment elevation myocardial infarction (STEMI) and non-ST segment elevation myocardial infarction (NSTEMI)) than those with angina pectoris (including unstable angina pectoris (UAP) and stable angina pectoris (SAP)) (64.51±3.32 *vs* 62.32±3.79, P<0.001). However, no significant difference about MPV, PDW, and P-LCR could be found in each group. In addition, inflammatory markers, including hs-CRP and Fbg, also elevated in STEMI and NSTEMI groups (P<0.001, P=0.007).

**Table 2:** MA_Thrombin_, platelet volume indices and inflammatory markers of study population according to clinical presentation

Characteristics	Overall	STEMI	NSTEMI	UAP	SAP	P-value
	N=206	N=67	N=50	N=33	N=56	
**Thrombelastography**						
MA_Thrombin_ (mm)	63.57±5.29	64.23±5.71	64.90±4.47	62.24±5.81	62.37±4.81	**0.026**
**Platelet volume indices**						
MPV(fL)	11.14±1.05	11.09±0.95	11.14±1.03	11.30±1.06	11.10±1.18	0.794
PDW(%)	13.60±2.29	13.44±2.04	13.70±2.27	13.96±2.38	13.49±2.57	0.706
P-LCR(%)	34.08±8.26	33.96±7.60	34.16±8.30	35.56±8.83	33.30±8.73	0.665
**Inflammatory markers**						
hs-CRP(mg/L)	10.68±19.34	13.46±19.22	18.94±28.04	5.60±11.57	3.00±5.03	**<0.001**
Fbg(mg/mL)	306.42±95.19	318.12±102.88	331.42±98.78	303.09±93.11	272.05±73.44	**0.007**

Data are expressed as mean ± standard deviation(SD).

STEMI, ST segment elevation myocardial infarction; NSTEMI, non-ST segment elevation myocardial infarction; UAP, unstable angina pectoris; SAP, stable angina pectoris; MPV, mean platelet volume; PDW, platelet distribution width; P-LCR, platelet-large cell ratio; hs-CRP, high-sensitivity C-reactive protein; Fbg, Fibrinogen.

### Correlation of MA_Thrombin_ and PVIs

Table 3 illustrates the relationship between MA_Thrombin_ and PVIs. Although we tried to analyze the association between MA_Thrombin_ and PVIs in each group, statistical correlation of these two kinds of biomarkers could only be found in NSTEMI patients. Since their link might interact with different confounders, three consecutive models of multivariate adjustment were created. The first model adjusted for age and sex, the second included lifestyle and chronic disease variables (smoking, BMI, diabetes, hypertension, dyslipidemia, stroke, WBC, platelet count, fasting blood glucose, and serum creatinine). Finally, we performed the third model taking medication history (aspirin, anti-coagulant medications, statins, β-blockers, and PPIs) into consideration. As shown in Table 3, MA_Thrombin_ and PVIs were still correlated in NSTEMI group after full adjustment by multiple linear regression analysis (MPV, r=0.393, P=0.007; PDW, r=0.334, P=0.023; P-LCR, r=0.382, P=0.008).

**Table 3:** Association between MA_Thrombin_ and platelet volume indices or inflammatory markers according to clinical presentation

	Overall (N=206)		STEMI (N=67)		NSTEMI (N=50)		UAP (N=33)		SAP (N=56)	
	Standardized correlation coefficient	P-value	Standardized correlation coefficient	P-value	Standardized correlation coefficient	P-value	Standardized correlation coefficient	P-value	Standardized correlation coefficient	P-value
*Platelet volume indices*										
MPV										
Model 1	0.038	0.567	−0.034	0.775	0.228	0.094	0.240	0.184	−0.073	0.560
Model 2	**0.218**	**0.001**	0.108	0.408	**0.388**	**0.002**	0.246	0.127	0.210	0.084
Model 3	**0.231**	**<0.001**	0.093	0.505	**0.393**	**0.007**	0.291	0.136	0.164	0.195
PDW										
Model 1	0.042	0.533	0.007	0.955	0.145	0.287	0.209	0.247	−0.058	0.643
Model 2	**0.229**	**0.001**	0.136	0.309	**0.344**	**0.009**	0.221	0.182	**0.271**	**0.031**
Model 3	**0.247**	**<0.001**	0.125	0.380	**0.334**	**0.023**	0.298	0.151	0.221	0.091
P-LCR										
Model 1	0.073	0.276	0.022	0.855	0.227	0.096	0.265	0.141	−0.064	0.615
Model 2	**0.252**	**<0.001**	0.143	0.275	**0.376**	**0.003**	0.268	0.097	**0.282**	**0.027**
Model 3	**0.260**	**<0.001**	0.137	0.325	**0.382**	**0.008**	0.309	0.110	0.232	0.080
*Inflammatory markers*										
hs-CRP										
Model 1	**0.274**	**<0.001**	**0.476**	**<0.001**	0.022	0.873	0.208	0.253	0.201	0.116
Model 2	**0.266**	**<0.001**	**0.485**	**<0.001**	−0.144	0.227	0.141	0.450	0.213	0.119
Model 3	**0.228**	**<0.001**	**0.499**	**<0.001**	−0.166	0.210	−0.005	0.983	**0.444**	**0.001**
Fbg										
Model 1	**0.507**	**<0.001**	**0.520**	**<0.001**	**0.449**	**0.001**	**0.552**	**0.001**	**0.622**	**0.000**
Model 2	**0.439**	**<0.001**	**0.482**	**<0.001**	**0.331**	**0.017**	**0.368**	**0.040**	**0.647**	**<0.001**
Model 3	**0.417**	**<0.001**	**0.500**	**<0.001**	**0.312**	**0.042**	0.278	0.182	**0.477**	**<0.001**

Values are expressed as standardized coefficients and P-value.

STEMI, ST segment elevation myocardial infarction; NSTEMI, non-ST segment elevation myocardial infarction; UAP, unstable angina pectoris; SAP, stable angina pectoris; MPV, mean platelet volume; PDW, platelet distribution width; P-LCR, platelet-large cell ratio; hs-CRP, high-sensitivity C-reactive protein; Fbg, fibrinogen.

Model 1: adjusted for age and sex.

Model 2: adjusted for age, sex, smoking, BMI, diabetes, hypertension, dyslipidemia, stroke, white blood cell count, platelet count, fasting blood glucose, and serum creatinine.

Model 3: adjusted for age, sex, smoking, BMI, diabetes, hypertension, dyslipidemia, stroke, white blood cell count, platelet count, fasting blood glucose, serum creatinine, aspirin application, current use of anti-coagulant medications, statins, β-blockers, and PPIs.

### Correlation of MA_Thrombin_ and inflammatory markers

Table 3 also illustrates the relationship between MA_Thrombin_ and hs-CRP. When multivariate analysis in subgroups were conducted, the correlation between the two biomarkers could be detected in STEMI and SAP patients upon full adjustment (STEMI, r=0.499, P<0.001; SAP, r=0.444, P=0.001). Meanwhile, MA_Thrombin_ also existed a positive correlation with Fbg in each group, independent of the CAD type (Table 3). Additionally, we analyzed the relationship between hs-CRP and Fbg and found that they were also correlated in all groups (P<0.001).

## DISCUSSION

To our knowledge, this is the first observational study to explore the potential relationship between magnitude of MA_Thrombin_ measured by TEG and PVIs along with inflammatory markers in different types of Chinese CAD patients. After adjusting for potential confounders (including age, sex, smoking, BMI, diabetes, hypertension, dyslipidemia, stroke, WBC, platelet count, fasting blood glucose, serum creatinine, aspirin, current use of anti-coagulant medications, statins, β-blockers, and PPIs), we found there was a trend that MA_Thrombin_ was tightly correlated to PVIs in patients with NSTEMI, but was more likely to have statistical correlation with hs-CRP in STEMI and SAP patients. In addition, MA_Thrombin_ was correlated to Fbg in all the patients enrolled.

Previous studies suggest that platelet not only participates in coronary thrombosis, but also contributes to atherosclerosis and endothelial injury by secreting mediators during the development of CAD [15, 16]. Some biomarkers of platelet activation may relate to the presentation of CAD clinically [5, 16, 17]. Up till now, MA_Thrombin_ measured by TEG has been shown to reflect the maximal potential aggregability of platelet, which, to some extent, can evaluate the contribution from platelet to coronary thrombosis specifically [5, 13, 18, 19]. Moreover, MA_Thrombin_ has been suggested as a predictor of adverse cardiac events after coronary stent implantation [13, 20, 21]. In this study, we revealed that the magnitude of MA_Thrombin_ was much higher in STEMI and NSTEMI patients than that in UAP and SAP patients, which partly supported the previous hypotheses and provided further evidence to the diagnostic and evaluating value of platelet aggregation assay in CAD patients.

Although many factors may confound the measurement of PVIs, several investigations have still demonstrated PVIs, such as MPV, PDW, and P-LCR, to be elevating in ACS and have hypothesized MPV to be an effective indicator of platelet activation *in vivo* [9–11, 22]. Our research didn't find statistical difference of PVIs among each group, which seemed to be controversial with previous studies [9–11, 22]. Inflammation plays a vital role in the formation, development, and disruption of atherosclerotic plaques, therefore, coronary atherosclerosis (AS) can also be regarded as an inflammatory process [4]. As a marker for inflammation, hs-CRP reflects a wide range of inflammatory conditions, which has been proved to be tightly associated with the occurrence and poor prognosis of CAD [12–14]. Fbg, as a coagulation factor, has also been recognized as an inflammatory marker as well. Our research showed serum hs-CRP and Fbg levels were higher in AMI patients than angina cases, in accordance with those earlier findings.

From the view of biomarkers, our study demonstrated that MA_Thrombin_, as a parameter of platelet aggregation, elevated in AMI patients, correlating to PVIs in NSTEMI group, but linking with inflammatory markers in STEMI group. These findings may provide new clinical evidences to the pathogenesis of platelet aggregation in AMI. Several probable mechanisms might have been proposed but the exact biologic mechanisms are not fully understood. NSTEMI is more likely to be caused by severe coronary lesions and repeated plague ruptures, which could induce platelet activation and may enhance platelet aggregating function in a relative long term. Cellular structure can determine but also can be influenced by function. Since larger platelets can contain and release more pro-aggregating mediators [9–11], PVIs might higher up to match this functional enhancement during the development of NSTEMI. In contrast, when STEMI occurs, coronary plaque rupture leads to platelet aggregating immediately, which contributes to the formation of coronary thrombus. Platelets adhere to vessels and release mediators that increase endothelial cell activation and leukocyte recruitment [23–25], as a result, expression of hs-CRP and Fbg are up-regulated rapidly. In the meantime, Fbg is an important factor of clotting system, it can play a key role in the blood clotting cascade. However, there is not enough time allowing PVIs to higher up during the acute phase. From another perspective, the development of atherosclerotic plaque can be regarded as a chronic inflammatory process. Plaque rupture may exacerbate the chronic inflammation acutely. We failed to find the association between MA_Thrombin_ and inflammatory markers in patients with NSTEMI or between MA_Thrombin_ and PVIs in patients with STEMI, which might also due to the probable machnisms mentioned above. In brief, NSTEMI is more likely to be a result of severe coronary stenosis in a relative long term rather than plaque rupture alone, so that, the correlation between MA_Thrombin_ and markers of acute phase reaction couldn't be detected sharply; however, STEMI are to the opposite.

As a cross-sectional study, our research might be affected by reverse causality and survivor bias. Several limitations should be acknowledged. First, this was a retrospective study and the study groups were of different sample sizes, moreover, baseline characteristics in each group were imbalanced. Second, although we have confirmed MA_Thrombin_ to be concerned with PVIs in NSTEMI and with inflammatory markers in STEMI, a prospective case-control study should be conducted to confirm these findings. Third, to clarify the exactly biologic mechanisms, further experimental investigations are needed. Moreover, the usage of clopidogrel or ticagrelor were not taken into baseline characteristics collection, which might skew the results potentially. Finally, other possibility of residual confounders related to these issues remains.

In conclusion, our data shows that both MA_Thrombin_ and inflammatory markers elevate in patients with AMI. Interestingly, MA_Thrombin_ is correlated to PVIs in NSTEMI but has inner-link with inflammatory markers in STEMI patients. which may suggest different mechanisms of platelet aggregation in these two types of AMI. Since MA_Thrombin_ not only can reflect the functional status of platelet, but also can reflect inflammation status indirectly, this marker may be used as a potential clinical parameter to evaluate platelet aggregation and inflammation together. Further investigations are necessary to confirm these findings.

## MATERIALS AND METHODS

### Study design and patients

From January to September 2015, 206 Chinese CAD patients admitted to Zhongshan Hospital, Fudan University were enrolled in this observational single centre study. Exclusion criteria were: older than 75 years of age, with a history of pulmonary artery embolism, deep venous thrombosis, peripheral arterial disease, anaemia, malignant disease, severe renal or hepatic insufficiency, total platelet count≤100×10^9/L, increased risk of bleeding or hematologic disorder, accepted thrombolytic therapy, usage of warfarin or new oral anticoagulant (NOAC), and missing data. The patients were grouped according to their clinical presentation [26, 27]. Finally, sixty-seven ST segment elevation myocardial infarction (STEMI) patients, fifty non-ST segment elevation myocardial infarction (NSTEMI) patients, thirty-three unstable angina pectoris (UAP) patients and fifty-six stable angina pectoris (SAP) patients were taken into consideration. All the subjects enrolled had been under dual anti-platelet therapy before they received coronary angiogram, which included aspirin 100 mg per day and clopidogrel 75 mg per day after each loading does of 300 mg. The study protocol was approved by the hospital's medical ethics committee, and informed consent was obtained from each patient.

### Blood samples

Blood samples were drawn by trained phlebotomists from the subjects before they received coronary angiogram. A complete blood cell count, including MPV, PDW, and PLC-R, was obtained by Sysmex XE-2100. Blood biochemical analysis including hs-CRP was tested by Hitachi 7600 autobiochemistry analyzer (Tokyo, Japan), while plasma level of Fbg was measured by Sysmex CA-1500 type automatic programming coagulation analyzer.

### Thromboelastography

TEG of citrated whole blood was performed using according to the manufacturer's instruction (Haemonetics, New York, USA). One mL whole blood was added into standard container coated with Kaolin (activator) and inverted five times. After 5 minutes resting, 340μL mixtures were loaded in a TEG cup containing 20μl of CaCl_2_. TEG was performed immediately and lasted from 15 minutes to 2 hours (depending on the blood samples) until MA_Thrombin_ readoutwas recorded. If patients were under anti-coagulation therapies by intravenous heparin or low molecular weight heparin during sample collection, heparinase coated TEG cups were used to generate TEG profiles.

### Assessment of demographic variables and other risk factors

Information on demographic and clinical characteristics (e.g. age, sex, smoking, and history of diseases and medication) were collected. Smoking was classified according to “current smoker or quitting less than one year”, or “nonsmoker or quitting more than one year”. BMIs were defined based on measured heights (accurate to 0.1cm) and weights (accurate to 0.1kg), and calculated as the body weight (kg) divided by the square of height (m^2^). The information of disease history included hypertension, diabetes, dyslipidemia, and stroke. Hypertension was based on a history of hypertension, or use of antihypertensive medication, or a systolic pressure≥140 mmHg, or a diastolic blood pressure≥90 mmHg. Diabetes was defined according to a self-reported history, current use of insulin or oral hypoglycemic agents, or fasting blood glucose level≥7.0 mmol/L. Dyslipidemia was diagnosed on the basis of a self-reported history, blood total cholesterol (TC) ≥5.18 mmol/L or triglycerides (TG) ≥1.7 mmol/L or low-density lipoprotein cholesterol (LDL-c) ≥3.37 mmol/L or high-density lipoprotein cholesterol (HDL-c)<1.04 mmol/L or current use of antilipemic agents. Medication history, including aspirin, current use of anti-coagulant medications (including intravenous heparin and low molecular weight heparin), statins, β-blockers, and PPIs were documented as “yes” or “no”. Levels of serum creatinine, fasting blood glucose, and other biomarkers were detected along with PVIs and inflammatory markers as mentioned above. Left ventricular ejection fraction (LVEF) were measured by ultrasonic cardiogram.

### Statistical analysis

Statistical analyses were performed using SPSS software, version 20.0 (SPSS Inc., Chicago, Illinois). Continuous variables were described by mean ± standard deviation (SD) and were analyzed using ANOVA analysis. Categorical variables were described by percentages and were compared using Chi-square tests. Multiple linear regression was used to determine the correlation among continuous variable in order to assess the association between MA_Thrombin_ and levels of PVIs and inflammatory markers with other parameters such as age, sex, hypertension, diabetes and BMI adjusted. All statistical tests were 2-sided, and *P*<0.05 was accepted as statistically significant.
